# Microbe and host interaction in gastrointestinal homeostasis

**DOI:** 10.1007/s00213-019-05218-y

**Published:** 2019-03-21

**Authors:** Rachael Horne, James St. Pierre, Sufian Odeh, Michael Surette, Jane A. Foster

**Affiliations:** 1 Department of Psychiatry and Behavioural Neurosciences, McMaster University, St. Joseph’s Healthcare, 50 Charlton Ave. E, T3308, Hamilton, ON L8N 4A6 Canada; 2 Department of Medicine, McMaster University, Hamilton, ON Canada; 3 Farncombe Family Digestive Health Research Institute, Hamilton, ON Canada; 4 Department of Psychiatry, St. Michael’s Hospital, Toronto, ON Canada

**Keywords:** Gut-brain axis, 16S rRNA gene sequencing, Antibiotics, Inbred strain, Gut barrier permeability

## Abstract

**Rationale:**

Researchers in psychiatry and neuroscience are increasingly recognizing the importance of gut-brain communication in mental health. Both genetics and environmental factors influence gut microbiota composition and function. This study examines host-microbe signaling at the gastrointestinal barrier to identify bottom-up mechanisms of microbiota-brain communication.

**Objectives:**

We examined differences in gut microbiota composition and fecal miRNA profiles in BALB/c and C57BL/6 mice, in relation to gastrointestinal homeostasis and evaluated the response to perturbation of the gut microbiota by broad-spectrum antibiotic treatment.

**Methods and results:**

Differences in the gut microbiota composition between BALB/c and C57BL/6 mice, evaluated by fecal 16S rRNA gene sequencing, included significant differences in genera *Prevotella*, *Alistipes*, *Akkermansia*, and *Ruminococcus*. Significant differences in fecal miRNA profiles were determined using the nCounter NanoString platform. A BLASTn analysis identified conserved fecal miRNA target regions in bacterial metagenomes with 14 significant correlations found between fecal miRNA and predicted taxa relative abundance in our dataset. Treatment with broad-spectrum antibiotics for 2 weeks resulted in a host-specific physiological response at the gastrointestinal barrier including a decrease in barrier permeability in BALB/c mice and alterations in the expression of barrier regulating genes in both strains. Genera *Parabacteroides* and *Bacteroides* were associated with changes in barrier function.

**Conclusions:**

The results of this study provide insight into how specific taxa influence gut barrier integrity and function. More generally, these data in the context of recent published studies makes a significant contribution to our understanding of host-microbe interactions providing new knowledge that can be harnessed by us and others in future mechanistic studies.

**Electronic supplementary material:**

The online version of this article (10.1007/s00213-019-05218-y) contains supplementary material, which is available to authorized users.

## Introduction

The gastrointestinal tract is home to trillions of microorganisms, which are important to host-pathogen defense, development of the immune system (Atarashi et al. 2011; Bouskra et al. 2008), energy metabolism (Backhed et al. 2004; Bouskra et al. 2008; Murphy et al. 2010), and maintenance of gastrointestinal homeostasis (Dowds et al. 2015; Smith et al. 2013). Under healthy conditions, the host and gut microbiota are in a state of homeostasis, forming a symbiotic and commensal relationship at the gastrointestinal barrier. Intestinal epithelial cells (IECs), secretory goblet cells, Paneth cells, and enteroendocrine cells (EECs) comprise a functional barrier between the gut luminal contents and the rest of the body. This interface is a primary site of communication between the gut microbiota and the host and is maintained by the expression and secretion of many critical proteins such as the those found in barrier regulating tight junctions, secretory IgA, mucus forming mucin, and antimicrobial peptides (Wells et al. 2017). In the past decade, the importance of gut microbiota to behavior and brain function has emerged as an important topic for neuroscience (Bharwani et al. 2016; Cryan and Dinan 2012; Dinan et al. 2013; Foster and McVey Neufeld 2013; Kelly et al. 2016; Tillisch et al. 2017). Studies using germ-free mice, raised in a sterile microisolator and lacking all microbes, have suggested that microbiota influence stress reactivity, neural function, neuroplasticity, neurogenesis, and behavior (Clarke et al. 2013; Heijtz et al. 2011; Luczynski et al. 2016; McVey Neufeld et al. 2013; Neufeld et al. 2011a, b). Additionally, exposure to broad-spectrum antibiotics that leads to an altered gut microbiota composition has been shown to influence CNS systems and behavior (Bercik et al. 2011; Delungahawatta et al. 2017; Frohlich et al. 2016; Hoban et al. 2016; Leclercq et al. 2017). A better understanding of host-microbe signaling at the gastrointestinal barrier is needed to identify bottom-up mechanisms of microbiota-brain communication. To that end, this study sought to identify key bacterial taxa that influence gut barrier function and to examine host-microbe signaling in untreated and antibiotic-treated mice.

Previous work has demonstrated that different inbred strains of mice have distinct gut microbiota composition; however, reports have also shown that the same strain of mice from different suppliers have distinct microbiota compositions (Benson et al. 2010; Brinkman et al. 2013; Campbell et al. 2012; Choo et al. 2017; Deloris et al. 2006; Ericsson et al. 2015; Hoy et al. 2015; Kovacs et al. 2011; O’Connor et al. 2014). Understanding the factors that influence inter-individual differences in gut microbiota composition and how these differences influence host physiology is a core area of interest in the microbiota-brain field. The current study examined baseline differences in gut microbiota composition in BALB/c and C57Bl/6 mice obtained from two suppliers, changes in bacterial taxa following exposure to broad-spectrum antibiotics, and how these changes influenced gastrointestinal barrier function. Based on recent work that suggested host-derived fecal miRNA may influence gut microbiota composition (Liu et al. 2016; Liu and Weiner 2016), we also examined fecal miRNA composition in samples from untreated BALB/c and C57Bl6 mice to explore the potential association of fecal miRNA and bacterial taxa.

## Methods

### Animals and experimental design

**Fig. 1:**
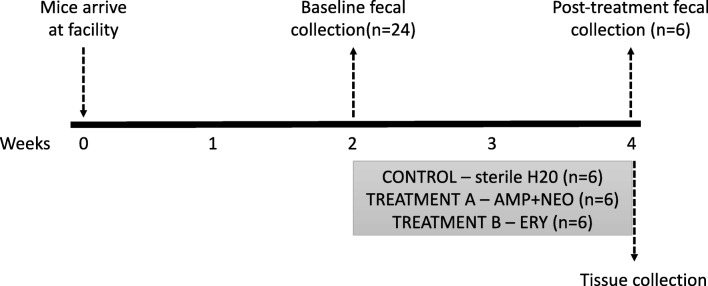
Experimental design. Female BALB/c and C57Bl/6 mice were acclimatized for 2 weeks to the facility. Antibiotics were administered in the drinking water for 2 weeks. Fecal samples were collected prior to administration and at the end of treatment. Tissue collection was conducted on the last treatment day

Female BALB/c and C57BL/6 mice were obtained from Charles River (St. Constant, Canada; Kingston, USA, respectively) at 8 weeks of age. The mice were maintained in specific pathogen-free housing in sanitized cages with filter bonnets, two per cage at St. Joseph’s Healthcare animal facility, under a 12 h light–12 h dark cycle, with lights on at 5 AM. At 10 weeks of age, the mice were identified by ear punch. The experimental design is provided in Fig. 1. Baseline fecal samples were collected from all mice (*n* = 24 per strain) and then mice were divided into treatment groups (*n* = 6 per treatment per strain); treatment A consisted of ampicillin 1 mg/ml, neomycin 2 mg/ml, primaricin 1.2 μg/ml (AMP + NEO); treatment B consisted of erythromycin 1 mg/ml, primaricin 1.2 μg/ml (ERY), and untreated control (CON) sterile water. A subset of mice were administered bacitracin (5 mg/ml), neomycin (5 mg/ml), and primaricin (1.2 μg/ml); however, due to significant weight loss, this treatment was terminated. Antibiotics were administered in the drinking water in a volume of 200 ml of sterile water, changed twice weekly. Food and water consumption data collection occurred twice weekly during cage maintenance. Two-minute handling took place at the same time. Fecal pellets were collected at the end of treatment. Pellets were placed in Eppendorf tubes, immediately frozen, and stored at − 80 °C until DNA/RNA extraction. All experimental procedures followed the guidelines of the Canadian Council on Animal Care and were approved by the Animal Research Ethics Board, McMaster University, Hamilton, Ontario, Canada.

### 16S rRNA sequencing and OTU processing

DNA was extracted from fecal pellets as previously described (Bartram et al. 2011; Whelan et al. 2014). Sequences of the 16S rRNA gene variable 3 (v3) region were amplified with a previously described procedure (Bartram et al. 2011) with the modifications of Whelan and colleagues (Whelan and Surette 2017; Whelan et al. 2014) and sequenced using the Illumina MiSeq platform. The MiSeq sequence reads were processed as described previously (Whelan et al. 2014). In summary, Cutadapt (Martin 2011) was utilized to trim reads, and sequences were aligned with PANDAseq (Masella et al. 2012). Operational taxonomic units (OTUs) were picked with AbundantOTU+ (Ye 2011) with a clustering threshold of 97% sequence similarity. Finally, the Ribosomal Database Project (RDP) classifier (Wang et al. 2007) assigns taxonomy up to the genus level using the Greengenes 2011 reference database (February 4, 2011 release; (DeSantis et al. 2006)). Using QIIME version 1.9.1 (Caporaso et al. 2010), singleton OTU’s were removed and the final OTU table generated for further analysis. Alpha diversity and beta diversity analyses were completed using the vegan package in R version 3.3.1 (R Core Team 2016). Analyses were done at the OTU level unless specified. An initial comparison of bacterial diversity between untreated BALB/c (*n* = 24) and C57BL/6 (*n* = 24) mice was completed to identify strain-related differences in bacterial taxa. A single sample from the C57BL/6 control group was removed due to sampling error.

### Fecal RNA isolation

RNA was extracted from fecal samples using Stool total RNA purification kit (#49400 Norgen Biotech Corporation, Thorold, ON) with the following modifications: fecal pellets were removed from − 80C storage and thawed on ice in lysis buffer for 10 min to rehydrate pellets; samples were manually homogenized to ensure the beads within the column had adequate access to the sample and to promote even homogenization and lysing of all cells. Samples were vortexed for 10 min. An additional wash step was added prior to eluting purified RNA to improve purity and to ensure contaminates were removed prior to NanoString sequencing as recommended by the manufacturer’s troubleshooting procedure. RNA yield was assessed by A260/A280 and A260/A230 ratios analyzed with a Nano-Drop® ND-1000 spectrophotometer (NanoDrop Technologies). RNA quality was accessed using Agilent 2100 Bioanalyzer with a eukaryote total RNA Nano assay and Agilent small RNA Kit for small RNA. The electropherograms were analyzed using the Agilent 2100 Expert Bioanalyzer Software.

### Quantitative NanoString nCounter fecal miRNA analysis

NanoString nCounter technology allows expression analysis of multiple genes from a single sample. nCounter® mouse miRNA v1.5 Assay Kit (NanoString Technologies) was used to detect miRNA in fecal RNA samples. Approximately 200 ng of total RNA was loaded in nCounter analysis following the manufacturer’s protocol. Data was processed and analyzed with nSolver™ Analysis Software 4.0 with background subtraction set to the average of the negative control reads + 2× SD of the negative control read and normalization to positive and ligation controls. Differential miRNA expression between BALB/c and C57BL/6 mice were visualized by volcano plot generated ggplot2 package in R version 3.3.4 (R Core Team 2016), significant differences were determined by *t* test. To compare miRNA expression profiles between groups, principal component analysis (PCA) performed using R package DESeq2, miRNA counts were transformed using regularized log (Love et al. 2014) to account of variation in counts across samples, top 50 variable miRNA were included in the PCA. PCA results were plotted used ggplot2. Abundant miRNA where determined by examining miRNA count data, miRNA probes with average counts > 24 above the background subtraction across all samples were considered abundant. A pathway analysis of differentially expressed miRNAs and abundant miRNAs was carried out with DIANA-miRPath v3.021 (Vlachos et al. 2015), using predicted microRNA targets from the DIANA-microT-CDS v5.0 algorithm and Gene Ontology gene sets derived from KEGG. The *p* value threshold was set to 0.05 and MicroT threshold to 0.8, multiple comparisons were corrected using FDR < 0.05.

### Predicting bacterial gene targets of abundant fecal miRNA

Abundant fecal miRNA sequences were used to explore potential bacterial gene targets of host miRNA by BLASTn alignment (Altschul et al. 1997) using a mouse gut microbial gene catalog database (Xiao et al. 2015) composed of 2,572,074 genes obtained from the GigaScience Database (http://gigadb.org). Bacterial genes that were selected by the BLASTn analysis were then matched to taxonomic classification using CARMA3 integrated US National Center for Biotechnology Information (NCBI-NR) database. To note, miRNA exist in a folded structure and the optimal binding regions on the mRNA transcript as determined by predicted secondary structure are not identical to the regions that were predicted using the BLASTn analysis. From a computation and bioinformatic standpoint, this is not unexpected as BLASTn only looks for sequence alignment (similarity) and does not take into account any secondary structure and extracts only the sequence alignments that had the most conserved bases with no gaps, often getting identities within range of 90–100% sequence coverage of the query miRNA. To address this limitation, we collected the free energy for the binding, and the duplex formation of the optimal sites as predicted by RNAup software, and also collected the free energy of binding to the regions that were predicted as per the BLASTn analysis (Supplemental XLS1). The relationship between the top expressed miRNAs and potential bacterial taxa targets identified was explored further in our dataset by assessing the Spearman’s rank correlation between fecal miRNA count data and the relative abundance of the selected taxa in compositional 16S RNA data from the same mice.

### Small intestinal permeability

The permeability of the small intestine was measured after antibiotic treatment via gavage of a fluorescent probe, unconjugated fluorescein isothiocyanate (FITC) (F1906; Invitrogen, Eugene, OR), and quantification of its recovery in serum. Mice were fasted (allowed water) for 3 h and weighed prior to gavage. Mice were administered 200 μl of 1.25 mg/ml FITC solution by gavage technique, mice were decapitated 3 h post-gavage, and blood and small intestinal tissue were collected. Blood collected at room temperature and then was centrifuged (10,000*G*, 10 min, ambient temperature) and serum collected. Fluorescence was measured for each serum sample in 96-well microtiter plates using SpectraMax Gemini EM microplate reader with excitation 485 nm and emission 528 nm (Molecular Devices, San Jose, CA). A standard curve for calculating serum recovery of FITC was obtained by diluting unconjugated FITC 1 mg/ml in water. Intestinal permeability is presented as the concentration of FITC in serum normalized to mouse body weight to the power of 10^4^ (mg/ml/g) × 10^4^.

### RNA extraction, cDNA generation, and gene expression analysis

Small intestinal tissue was analyzed by qRT-PCR for expression of tight junction protein mRNAs including occludin (Ocln), claudin 7 (Cldn7), zonula occluden-1 (ZO-1), and mucus-related mucin-2 (Muc-2) mRNA. Total RNA was extracted using Norgen Biotek Animal Tissue RNA Purification kit according to the manufacturer’s protocol (Norgen Biotek Corp, Thorold Ontario, Canada). Isolated RNA samples were treated using DNA-free™ DNase kit (Ambion) as per manufacturer guidelines. RNA quality and yield were assessed by A260/A280 and A260/A230 ratios analyzed with a Nano-Drop® ND-1000 spectrophotometer (NanoDrop Technologies). A total of 1 μg of RNA was transcribed to cDNA in 20 μl reaction using Superscript III reverse transcriptase kit as per manufacturer’s protocol (Invitrogen), no reverse transcriptase reactions were included to confirm no DNA contamination. PCR amplification efficiency was determined for each primer pair target and reference gene used by generating calibration curves with a tenfold dilution of cDNA. Amplification efficiency was determined from the slope of the log-linear portion of the calibration curve for each gene tested. The relative gene expression of Ocln, Cldn7, ZO-1, and Muc-2 mRNAs was determined by RT-PCR using SsoAdvanced Universal SYBR Green Supermix (Bio-Rad), the amplification reactions were carried out in a CXF96 Real-Time Detection system (Bio-Rad). The comparative ddCt− method was used to determine the amount of target gene normalized to endogenous references (β-actin and GAPDH) and relative to a calibrator in the untreated control group. The purity of the PCR products was verified by melting curves and gel electrophoresis.

### Statistical analysis

Individual-based rarefaction curves were produced to account for differences in sequencing depth between 16S samples. Individual samples were rarefied 10 times at multiple depths up to the minimum sequencing depth of all samples in the analysis. The mean diversity of all 10 rarefactions was taken as the diversity measure for each sample at each depth. *t* tests were used to examine strain differences at various depths. Diversity metrics used in the analysis included the Chao1 index and the Shannon index. Beta diversity between samples was explored using principal coordinate analysis (PCoA) with Bray-Curtis, and binary (presence-absence) Bray-Curtis distance metrics were applied to rarefied OTU count data. Multiple rarefaction PCoA was used for all metrics, as differences in sequencing depth between samples has been shown to affect distance metrics that are sensitive to rare taxa (Weiss et al. 2017). PCoA was done separately on 100 random rarefactions at a depth of 11,353. Median coordinates, interquartile range of coordinates, and median variance explained across all 100 analyses were used to create PCoA plots. Significant baseline strain differences were assessed with PERMANOVA, using 100,000 permutations and the distance metrics above.

Permutation tests were used to assess differential abundance in bacterial composition between C57BL/6 and BALB/c mice at baseline (*n* = 24 per strain)). Analyses utilized relative taxa abundances of the unrarefied OTU table collapsed to the genus level. Median taxon abundance differences between strains, median change pre to post were used as effect size estimates to add interpretability to the results. Permutation-based repeated measures factorial ANOVA was used to assess the effects of antibiotic treatment over time and how these effects differed between mouse strains (*n* = 6 per strain per treatment). Analyses were run separately on the abundances of each taxon. The statistical method was implemented using the multi-stratum analysis function aovp in the lmPerm package (setting Ca = 0.000001 and maxIter = 100,000). All treatment groups, including controls, were included in the analysis to isolate effects that are specifically caused by antibiotics and not due to random shifts in microbiota composition over time. Individual mice were used as a blocking factor, allowing only permutations between pre- and post-treatment samples within individuals. Interactions of time with treatment and the three-way interaction of treatment by strain by time were assessed to find main effects of antibiotic treatment and differential effects of treatment between strains, respectively. For each mouse strain, separate post hoc repeated measures *t* tests within treatment groups were conducted to explore significant treatment effects and significant treatment by strain interactions from the above analysis. The Benjamin Hochberg procedure was used to correct for FDR across all taxa included in these analyses.

FITC recovery treatment and strain effects were assessed with permutation ANOVA and post hoc permutation *t* tests (perm.t.test in the Deducer package with 100,000 permutations). *p* < 0.05 was taken as the significance level. Correlations of bacterial abundance after treatment with FITC recovery were analyzed using Spearman’s rank-based correlation tests. A targeted analysis included only taxa with significant treatment effects or significant treatment by strain interactions in the above repeated measures analyses. To account for baseline strain differences in permeability, FITC recovery values for BALB/c and C57BL/6 mice in treatment AMP + NEO and ERY were transformed to *z* score distances away from respective control group means for each strain. These FITC *z* scores are representative of the change in FITC recovery compared to control levels for each mouse strain. An FDR of 0.05 was taken as significant among results from the targeted taxon list. Differences between treatment groups and the control groups’ gene expression were assessed by the unpaired *t* test or the non-parametric Mann-Whitney test which was used to assess Cldn7 due to non-normality. Correlation between gene expression and intestinal permeability were measured using Pearson correlation, and the correlation between gene expression and bacterial relative abundance were assessed using Spearman correlation.

## Results

### Differences in microbiota composition and diversity between BALB/c and C57BL/6 mice

**Fig. 2:**
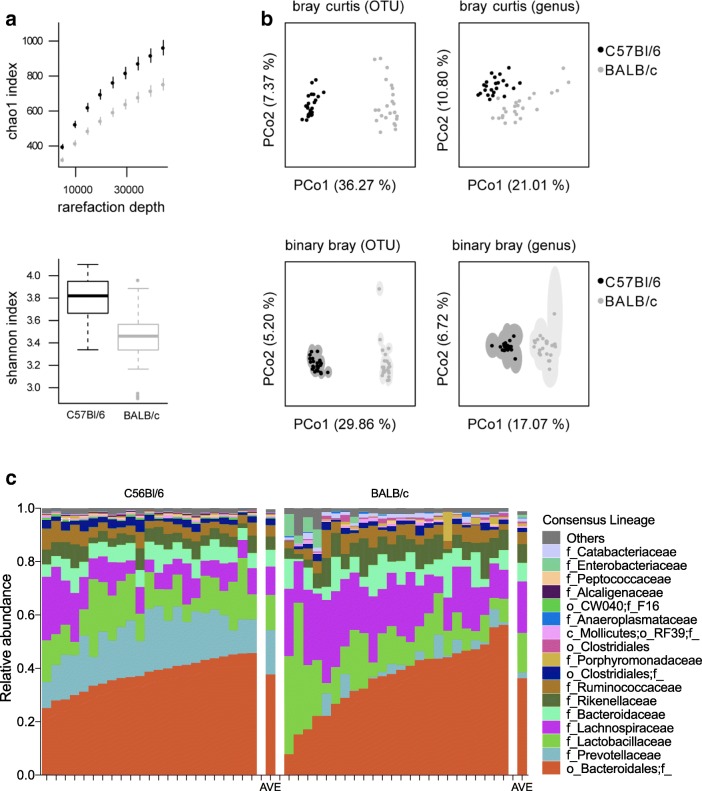
16S rRNA sequencing of BALB/c and C57BL/6 mice revealed host genetic differences in bacteria composition at the OTU and genus levels. **a** Alpha diversity using Chao 1 and Shannon indexes. **b** Beta diversity analysis, a principle coordinate analysis (PCoA) was done using the Bray-Curtis distance metric at the genus and OTU levels. Presence-absence (binary) Bray-Curtis distances were examined to display differences in rare taxa. PCoA was done separately on 100 rarefactions with points representing median coordinates and ellipses representing interquartile ranges across all 100 analyses. Median percent variance explained across rarefactions for principal coordinates plotting are also shown. **c** Relative abundance of bacterial taxa > 1% classified at the family-level taxonomy. Plots shown for individual mice (*n* = 24 BALB/c, *n* = 24 C57BL/6) and average relative abundance for BALB/c and C57BL/6 mice

Microbial compositions of fecal samples collected from healthy BALB/c and C57BL/6 mice (*n* = 24 per group) were analyzed by 16S rRNA sequencing. Sequencing data resulted in 5060 different OTUs belonging to 207 different assigned taxonomies, with a minimum and a maximum number of reads per sample of 11,353 and 139,193, and a median of 71,380 reads. Baseline differences in alpha and beta diversity were observed between BALB/c and C57BL/6 (Fig. 2). BALB/c mice were found to have significantly lower alpha diversity than C57BL/6 mice (Chao1: *p* < 0.05, Shannon: *p* < 0.05 at all rarefaction depths larger than 1000; Fig. 2). Samples were also found to cluster significantly by mouse strain in PCoA plots (PERMANOVA: median *p* < 0.05 for all metrics; Fig. 2c–e). Bray-Curtis distances were calculated using both untransformed and presence-absence (binary) abundance tables at the OTU and genus levels. This approach provided a visualization of diversity based on abundant and rare taxa. Bray-Curtis distance is more sensitive to changes in highly abundant taxa and less sensitive to rare taxa, while binary Bray-Curtis distances are equally sensitive to all taxa. The observed strain-specific clustering across all analyses visualizes strain-related differences in both abundant and rare OTUs.

The relative abundance of bacterial taxa in BALB/c and C57BL/6 mice at the family level is shown in Fig. 2c. Inter-individual variability in taxa was evident as shown in the abundance plots of individual mice. Average relative abundance plots are also provided (Fig. 2c). Permutation *t* tests revealed several taxa that differed in abundance between C57BL/6 and BALB/c mice (Table 1). At the genus level, 22 taxa were found to have a significantly higher abundance in C57BL/6 mice comparison to BALB/c with the largest difference in abundance found to be *Prevotella*, as well as higher abundances of *Akkermansia* and *Ruminococcus*. Five taxa were uniquely found in C57BL/6 mice including *Allobaculum*. In comparison, a total of 26 taxa at the genus level were found to have significantly higher abundance in BALB/c mice compared to C57BL/6 mice (Table 1), with only 3 taxa detected as unique to BALB/C mice. Of the 26 taxa found to be elevated in BALB/c mice, 17 belonged to the phyla Firmicutes, with families Lachnospiraceae, Catabacteriaceae, and Ruminococcaceae accounting for the largest differences in abundance. This is interesting as previous work showed Firmicutes taxa as a major contributor to microbiota differences in different mouse strains (O’Connor et al. 2014). Additionally, at the genus level, *Alistipes* and *Oscillospira* were found to have significantly higher abundance in BALB/c mice. BALB/c mice were found to have increased inter-individual variability in compositions at the genus level compared to C57BL/6 mice. This was demonstrated by the increasing spread and interquartile range (IQR) of BALB/c samples in PCoA plots (Fig. 2b) and in the increased variability of BALB/c compositions at higher phylogenetic levels (Fig. 2c). At the OTU level, BALB/c mice had increased inter-individual variability using the Bray-Curtis distance, but not using binary Bray-Curtis distance. This suggests that within C57BL/6 and BALB/c mice, consistent OTU’s are present, while the abundances of these OTU’s shift more between BALB/c mice than between C57BL/6 mice.

**Table 1:** Significant baseline relative abundance differences between C57BL/6 and BALB/c mice

Consensus.lineage	Difference
p_Bacteroidetes;c_Bacteroidia;o_Bacteroidales;f_Prevotellaceae;g_Prevotella	↑↑↑ C57BL/6
p_Bacteroidetes;c_Bacteroidia;o_Bacteroidales	↑ C57BL/6
p_Bacteroidetes;c_Bacteroidia;o_Bacteroidales;f_Rikenellaceae;g_	↑ C57BL/6
p_Cyanobacteria;c_4C0d-2;o_YS2;f_;g_	↑ C57BL/6
p_Firmicutes;c_Bacilli;o_Turicibacterales;f_Turicibacteraceae;g_	↑ C57BL/6
p_Firmicutes;c_Clostridia;o_Clostridiales;f_;g_	↑ C57BL/6
p_Firmicutes;c_Clostridia;o_Clostridiales;f_Lachnospiraceae;g_Ruminococcus	↑ C57BL/6
p_Firmicutes;c_Clostridia;o_Clostridiales;f_Peptococcaceae;g_	↑ C57BL/6
p_Firmicutes;c_Clostridia;o_Clostridiales;f_Ruminococcaceae	↑ C57BL/6
p_Firmicutes;c_Clostridia;o_Clostridiales;f_Ruminococcaceae;g_Subdoligranulum	↑ C57BL/6
p_Proteobacteria;c_Alphaproteobacteria;o_;f_;g_	↑ C57BL/6
p_Proteobacteria;c_Betaproteobacteria;o_Burkholderiales;f_Alcaligenaceae;g_	↑ C57BL/6
p_Tenericutes;c_Erysipelotrichi;o_Erysipelotrichales;f_Erysipelotrichaceae	↑ C57BL/6
p_TM7;c_TM7–3;o_CW040;f_F16;g_	↑ C57BL/6
p_Verrucomicrobia;c_Verrucomicrobiae;o_Verrucomicrobiales;f_Verrucomicrobiaceae;g_Akkermansia	↑ C57BL/6
p_Firmicutes;c_Clostridia;o_Clostridiales;f_Lachnospiraceae;g_Roseburia	(↑) C57BL/6
p_TM7;c_TM7–3;o_CW040	(↑) C57BL/6
p_Actinobacteria;c_Actinobacteria;o_Coriobacteriales;f_;g_	Only C57BL/6
p_Cyanobacteria;c_Oscillatoriophycideae;o_Chroococcales;f_Prochloraceae;g_	Only C57BL/6
p_Firmicutes;c_Clostridia;o_Clostridiales;f_ClostridialesFamilyXIII.IncertaeSedis;g_	Only C57BL/6
p_Proteobacteria;c_Alphaproteobacteria	Only C57BL/6
p_Tenericutes;c_Erysipelotrichi;o_Erysipelotrichales;f_Erysipelotrichaceae;g_Allobaculum	Only C57BL/6
p_Bacteroidetes;c_Bacteroidia;o_Bacteroidales;f_Rikenellaceae;g_Alistipes	↑↑ BALB/c
p_Firmicutes;c_Clostridia;o_Clostridiales;f_Catabacteriaceae;g_	↑↑ BALB/c
p_Firmicutes;c_Clostridia;o_Clostridiales;f_Lachnospiraceae	↑↑ BALB/c
p_Firmicutes;c_Clostridia;o_Clostridiales;f_Lachnospiraceae;g_	↑↑ BALB/c
p_Firmicutes;c_Clostridia;o_Clostridiales;f_Ruminococcaceae;g_Oscillospira	↑↑ BALB/c
p_Actinobacteria;c_Actinobacteria;o_Actinomycetales	↑ BALB/c
p_Actinobacteria;c_Actinobacteria;o_Actinomycetales;f_Corynebacteriaceae;g_Corynebacterium	↑ BALB/c
p_Actinobacteria;c_Actinobacteria;o_Coriobacteriales;f_Coriobacteriaceae	↑ BALB/c
p_Actinobacteria;c_Actinobacteria;o_Coriobacteriales;f_Coriobacteriaceae;g_Slackia	↑ BALB/c
p_Firmicutes;c_Bacilli;o_Lactobacillales;f_Carnobacteriaceae;g_	↑ BALB/c
p_Firmicutes;c_Bacilli;o_Lactobacillales;f_Streptococcaceae;g_Lactococcus	↑ BALB/c
p_Firmicutes;c_Clostridia;o_Clostridiales	↑ BALB/c
p_Firmicutes;c_Clostridia;o_Clostridiales;f_Clostridiaceae;g_	↑ BALB/c
p_Firmicutes;c_Clostridia;o_Clostridiales;f_ClostridialesFamilyXIII.IncertaeSedis;g_Eubacterium	↑ BALB/c
p_Firmicutes;c_Clostridia;o_Clostridiales;f_Lachnospiraceae;g_Anaerostipes	↑ BALB/c
p_Firmicutes;c_Clostridia;o_Clostridiales;f_Lachnospiraceae;g_Blautia	↑ BALB/c
p_Firmicutes;c_Clostridia;o_Clostridiales;f_Lachnospiraceae;g_Oribacterium	↑ BALB/c
p_Firmicutes;c_Clostridia;o_Clostridiales;f_Ruminococcaceae;g_Anaerotruncus	↑ BALB/c
p_Firmicutes;c_Clostridia;o_Clostridiales;f_Ruminococcaceae;g_Ethanoligenens	↑ BALB/c
p_Proteobacteria;c_Gammaproteobacteria;o_Enterobacteriales;f_Enterobacteriaceae;g_Escherichia	↑ BALB/c
p_Proteobacteria;c_Gammaproteobacteria;o_Pseudomonadales;f_Moraxellaceae;g_Moraxella	↑ BALB/c
p_Tenericutes;c_Erysipelotrichi;o_Erysipelotrichales;f_Erysipelotrichaceae;g_	↑ BALB/c
p_Tenericutes;c_Mollicutes;o_Anaeroplasmatales;f_Anaeroplasmataceae;g_Anaeroplasma	↑ BALB/c
p_Firmicutes;c_Bacilli;o_Bacillales;f_Bacillaceae;g_	Only BALB/c
p_Firmicutes;c_Bacilli;o_Bacillales;f_Bacillaceae;g_Bacillus	Only BALB/c
p_Firmicutes;c_Clostridia;o_Clostridiales;f_ClostridialesFamilyXI.IncertaeSedis;g_	Only BALB/c

↑↑↑, difference between medians is > 10%; ↑↑, > 1%; ↑, < 1%; (↑), increased presence

### Identification of murine fecal miRNA profile

**Fig. 3:**
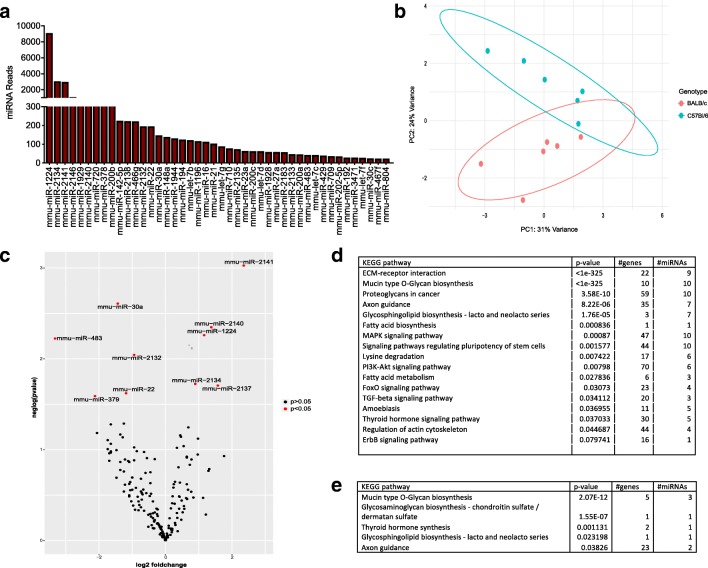
Host genetic related difference in expression of murine fecal miRNA. **a** Mean values for the 24 most abundant miRNAs in mouse fecal sample collected from both BALB/c (*n* = 6) and C57BL/6 (*n* = 6). **b** Principle component analysis (PCA) plot of fecal miRNA counts of top 50 variable miRNA. **c** Volcano plot of fecal miRNA detected by NanoString, BALB/c versus C57BL/6, *x*-axis log2 fold change of expression level between fecal miRNA from BALB/c and C57BL/6 mice, *y*-axis log10 *p* value from unequal variances *t* test between the compared groups. **d**, **e** Pathway analysis murine fecal miRNA performed using DIANA-miRPath v3.021, with predicted miRNA targets derived from MicroT-CDS v5.0, and Kyoto Encyclopedia of genes and genomes (KEGG) biological pathways. **c** Pathways of top 24 expressed murine miRNA excluded mmu-miR-2134, mmu-miR-2141, mmu-miR-2146, mmu-miR-2140, mmu-miR-720, mmu-miR-2138, mmu-miR-2132, mmu-miR-1944, mmu-miR-1196, mmu-miR-2137, mmu-miR-2135, and mmu-miR-2133. **d** Pathway analysis of differential expressed fecal miRNA BALB/c v C57BL/6

A comprehensive profile of fecal miRNA expression was generated using the nCounter NanoString mouse miRNA v1.5 assay. Of the 599 miRNA probes tested 75 were found to be detectable in fecal samples from CON Balb/C and CON C57Bl/6 mice (*n* = 6 untreated CON, collected at end of experiment) and 44 of those detected were considered abundant (> 24) with mmu-miR-1224, mmu-miR-2134, mmu-miR-2141, mmu-miR-2146, mmu-miR-1929, mmu-miR-2140, and mmu-miR-720 being the most abundant miRNA (Fig. 3a). A principle component analysis was performed to investigate the effect of host strain on fecal miRNA profiles and showed separation and clustering of miRNAs by host strain (Fig. 3b). Direct comparison of fecal miRNA expression between BALB/c and C57BL/6 mice revealed differentially expressed miRNA (Fig. 3c), with miR-2141, miR-2140, miR-1224-5p, and miR-30a identified with the greatest difference (*p* < 0.005).

### The functionality of fecal miRNA

The functional impact of the detected fecal miRNAs on the host biological pathways was assessed using an in silico analysis DIANA-miRPath v3 (Vlachos et al. 2015). Using DIANA-miRPath, we evaluated all the significantly targeted pathways by the differentially expressed and abundant fecal microRNAs (Fig. 3d, e). Of the 10 differentially expressed miRNAs, mir-2141, miR-2140, miR-2134, and miR-2132 were excluded from the pathway analysis as they are not currently annotated in miRbase (Griffiths-Jones et al. 2008). Mucin-type O-glycan biosynthesis pathway was found to be the most significantly affected by the differentially expressed miRNA. This pathway is responsible for the glycosylation of mucin, a ubiquitous intestinal glycoprotein that makes up the mucus layer which adheres to the intestinal epithelial cells on the lumen side of the gastrointestinal barrier (Bergstrom and Xia 2013). Significantly targeted pathways for the abundant miRNA across both strains included complex glycoprotein synthesis, with targets in the extracellular matrix (ECM) including collagen, integrins, lamins, acetyl-galactoaminyltransferases, chondroitin sulfates synthases, and fucosyltransferase 9 in addition to mucin glycan biosynthesis. This result is interesting in the context of host-microbe relationship as the ECM is highly dynamic and plays a large role in maintaining intestinal homeostasis, with its constant remodeling critical to the intestinal epithelium responsiveness to host-derived and microbial signals (Vllasaliu et al. 2014).

### Host fecal miRNA target bacterial genes

The association of fecal murine miRNA sequences and known bacterial genes was evaluated using a publicly available mouse gut metagenome (Xiao et al. 2015). Bacterial genes within the mouse gut metagenome were evaluated as potential fecal miRNA targets by nucleic acid sequence homology. Bacterial genes lack introns; therefore, alignment of miRNA sequence to a bacterial gene is predictive of miRNA-mRNA complementary to the bacterial mRNA transcript. The BLASTn alignment of the 44 top expressed miRNA sequences against the 2,572,074 genes in the gut microbial gene catalog resulted in 991 significant alignments (Supplemental XLS2). Of these 991 alignments, 11 were mapped to genes classified at the species level, 91 at the genus level and 107 at the family level. Of the total 991 alignments, 335 did not have a taxonomic classification associated with the targeted gene as only 67.8% of the total genes in the metagenome database have a taxonomic classification. To explore the putative association between fecal miRNA expression and gut bacteria, the association of the relative abundance of predicted taxa and level of miRNA in our fecal samples was assessed. Fourteen significance correlations between 13 miRNA abundances and their metagenomic-alignment-predicted taxa were observed, including significant positive correlations with *Escherichia* (Spearman’s *ρ* = 0.79, *p* < 0.01), *Akkermansia* (Spearman’s *ρ* = 0.65, *p* < 0.05), and *Staphylococcus* (Spearman’s *ρ* = 0.6, *p* < 0.05) and significant negative correlations with *Parabacteroides* (Spearman’s *ρ* = − 0.77, − 0.82, *p* < 0.001), *Prevotella* (Spearman’s *ρ* = − 0.66, *p* < 0.05), and *Clostridium* (Spearman’s *ρ* = − 0.63 to −0.77, *p* < 0.05 to 0.01; Table 2).

**Table 2:** Potential miRNA target taxa significantly correlate with miRNA abundance (genus-level and OTU-level analysis results)

miRNA	miRNA-16S data correlation analysis		miRNA-metagenome database BLASTn alignment			
	Taxa	Spearman *ρ* (*p* value)	Potential bacterial gene target	eggNOG3 annotation	Score (bit)	*E* value
mmu-miR-2141	*Escherichia*	0.79 (0.006)	11_GL0122160	NOG242382	30.2	4.6
mmu-miR-21	*Akkermansia*	0.65 (0.031)	10_GL0030843	NOG77418	30.2	7.6
mmu-miR-2140	*Staphylococcus*^a^	0.60 (0.049)	2A-dyr14-07_GL0001188	COG1109	30.2	4.6
mmu-let-7b	*Parabacteroides*^b^	− 0.82 (0.004)	2A-dyr13-06_GL0013439	NA	32.2	1.9
mmu-let-7c	*Parabacteroides*^b^	− 0.77 (0.008)	2A-dyr13-06_GL0013439	NA	30.2	7.6
mmu-miR-2134	*Prevotella*	− 0.66 (0.031)	16_GL0035169	NA	30.2	4.6
mmu-miR-22	B. *Bacteroides*_37^c^	0.67 (0.023)	10_GL0041934	NOG136220	30.2	7.6
mmu-miR-1944	B. *Bacteroides*_37^c^	0.64 (0.035)	G1-6A_GL0094981	COG0584	34.2	0.98
mmu-miR-1944	B. *Bacteroides*_40^c^	− 0.71 (0.014)	G1-6A_GL0094981	COG0584	34.2	0.98
mmu-miR-192	B. *Bacteroides*_40^c^	− 0.67 (0.024)	10_GL0073986	NA	30.2	6.1
mmu-miR-194	B. *Bacteroides*_25^c^	− 0.66 (0.031)	10_GL0012378	COG3015	32.2	1.9
mmu-miR-22	B. *Bacteroides*_40^c^	− 0.63 (0.036)	10_GL0041934	NOG136220	30.2	7.6
mmu-miR-16	C. *Clostridium*	− 0.77 (0.005)	G1-5A_GL0185204	NA	30.2	7.6
mmu-miR-16	C. *Clostridium*	− 0.77 (0.005)	S-Fe12_GL0134416	NA	30.2	7.6
mmu-miR-16	C. *Clostridium*	− 0.77 (0.005)	S-Fe20_GL0097563	NA	30.2	7.6
mmu-miR-200c	C. *Clostridium*	− 0.77 (0.005)	Group2-8A_GL0215737	COG0726	30.2	9.1
mmu-miR-200c	C. *Clostridium*	− 0.77 (0.005)	MH-0-5_GL0117732	COG3209	36.2	0.15
mmu-miR-1196	C. *Clostridium*	− 0.73 (0.010)	2A-dyr13-06_GL0004680	COG0673	32.2	1.5
mmu-miR-1196	C. *Clostridium*	− 0.73 (0.010)	35_GL0040992	COG0577	30.2	6.1
mmu-miR-1196	C. *Clostridium*	− 0.73 (0.010)	S-Fe9_GL0022151	NA	30.2	6.1
mmu-let-7d	C. *Clostridium*	− 0.68 (0.021)	6-2_GL0008179	NOG26044	30.2	7.6
mmu-let-7d	C. *Clostridium*	− 0.68 (0.021)	6-7_GL0078312	NOG26044	30.2	7.6
mmu-miR-21	C. *Clostridium*	− 0.68 (0.022)	S-Fe3_GL0200235	NA	30.2	7.6
mmu-miR-1944	C. *Clostridium*	− 0.67 (0.024)	1-3_GL0087789	NA	32.2	3.9
mmu-miR-1944	C. *Clostridium*	− 0.67 (0.024)	7-3_GL0015756	NA	32.2	3.9
mmu-miR-1944	C. *Clostridium*	− 0.67 (0.024)	S-Fe16_GL0006390	NA	32.2	3.9
mmu-let-7g	C. *Clostridium*	− 0.66 (0.027)	S-Fe11_GL0093182	NA	30.2	7.6
mmu-miR-23a	C. *Clostridium*	− 0.63 (0.038)	1A-dyr2-07_GL0021636	COG0714	30.2	6.1
mmu-miR-23a	C. *Clostridium*	− 0.63 (0.038)	Group2-5A_GL0024298	NOG39127	30.2	6.1
mmu-miR-23a	C. *Clostridium*	− 0.63 (0.038)	S-Fe9_GL0008839	NA	30.2	6.1

*B*. *Bacteroides*, Bacteroidaceae *Bacteroides*; *C*. *Clostridium*, Clostridiaceae *Clostridium*

^a^Alignment was to a metagenomics read classified to Staphylococcaceae. As *Staphylococcus* was the only genus found from the Staphylococcaceae family in the 16S data set, correlations with the aligned miRNA were done at the genus level

^b^Alignment was to a metagenomics read classified to Porphyromonadaceae. As *Parabacteroides* was virtually the only genus found from the Porphyromonadaceae family in the 16S data set (mean ± std; Porphyromonadaceae proportion, 1.00 ± 0.0005), correlations with the aligned miRNA were done at the genus level

^c^Alignment was to a metagenomics read classified to a species of *Bacteroides*. 16S correlations with the miRNA were done at the OTU level

### Antibiotic treatment shifts in microbiota composition

**Fig. 4:**
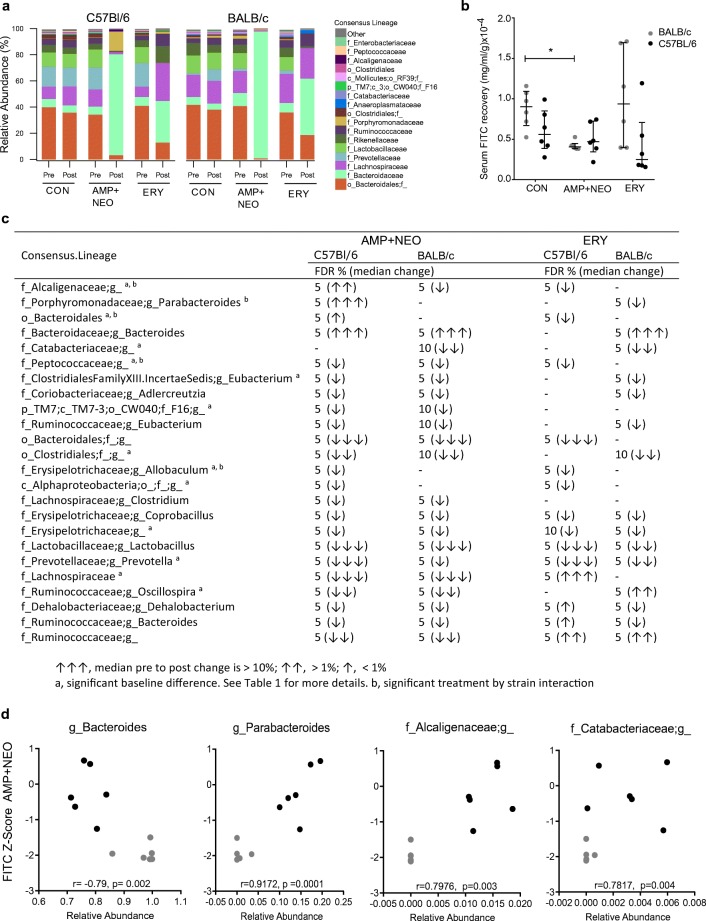
Impact of antibiotic treatment on gut microbiota composition and gastrointestinal barrier integrity. (**a**) Relative abundance of bacterial taxa classified to the family-level taxonomy pre- and post-treatment with antibiotics: ampicillin + neomycin (AMP + NEO) and erythromycin (ERY) for in BALB/c and C57BL/6 mice. (**b**) Small Intestinal epithelial permeability to fluorescein isothiocyanate (FITC), of control group (CON) C57BL/6 and BALB/c and permeability post antibiotic treatment AMP + NEO and ERY, *p* < 0.05. (**c**) Significant treatment effects on relative abundance at 16S genus level. (**d**) Changes in small intestinal permeability post antibiotic treatment with AMP + NEO correlate with bacterial taxa relative abundance post treatment. Spearman correlation of FITC *z* score to relative abundance of genera *Bacteroides* genus, unclassified *Catabacteriaceae*, *Parabacteroides*, and unclassified *Alcaligenaceae* genus

Antibiotic treatment significantly reduced the number of taxa and their overall distribution in both C57BL/6 and BALB/c mice as shown by the reduction in alpha diversity post treatment (Suppl. Fig. 1). Comparison of bacterial composition before and after antibiotic treatment revealed a significant interaction between treatment and time in both Bray-Curtis and binary Bray-Curtis analysis. Antibiotic treatments shifted microbiota compositions in both mouse strains, as seen in family level bar plots (Fig. 4a). An interaction between strain, treatment, and time was significant at the genus and OTU level using binary Bray-Curtis distance and at the OTU level using untransformed Bray-Curtis. This three-way interaction between strain, treatment, and time point indicates that antibiotic treatment differentially affected BALB/c and C57BL/6 microbiota compositions. No strain by treatment by time point interaction was found with untransformed Bray-Curtis at the genus level. These results suggest that both antibiotic treatments affected dominant genus-level taxa similarly in C57BL/6 and BALB/c mice. The results of the permutation-based repeated measures factorial ANOVA analysis done at the genus level confirm the composition-wide analysis, finding large shifts in the same direction in taxa affected by antibiotics in both C57BL/6 and BALB/c mice and small shifts in taxa affected in only C57BL/6 or BALB/c mice (Fig. 4d).

*Prevotella*, *Lactobacillus*, *Coprobacillus*, and Erysipelotrichaceae were the only taxa identified to significantly decrease in response to both antibiotic treatments independent of host mouse strain. An unidentified genus member of the Ruminococcacea family exhibited a consistent decrease in response to AMP + NEO treatment for both strains but an increase in response to ERY. There were 5 taxa identified with a significant strain interaction in response to treatment, including *Parabacteroides*, *Peptococcaceae*, an unclassified member of the Bacteroidales order, *Allobaculum*, and *Alcaigenanceae*. For *Parabacteroides*, *Peptococcaceae*, and an unclassified member of the Bacteroidales order, the largest response occurred in C57BL/6 mice. The other two detected interactions found within abundances of *Allobaculum* and *Alcaligenaceae* were primarily driven by the difference in abundance of these bacteria in BALB/c mice prior to treatment. Five identified taxa, *Eubacterium* (incertae sedis), *Dehalobacterium*, *Bacteroides* (Ruminococcaceae), *Adlercreutzia*, and *Eubacterium* (Ruminococcaceae) also responded differentially by host mouse strain to treatment though non-significant interactions after correcting for FDR.

### Disruption of barrier in AMP + NEO-treated BALB/c mice small intestine permeability

Small intestinal permeability was accessed by the recovery of paracellular fluorescent probe FITC in serum. A permutation ANOVA of FITC recovery found a significant main effect of strain (*p* < 0.05) and an interaction of strain by treatment that tended toward significance (*p* < 0.1; Fig. 4b). Treatment significantly altered gut permeability in the BALB/c mice (permutation ANOVA: *p* < 0.05), but not C57BL/6 mice (permutation ANOVA: *p* > 0.1). Specifically, BALB/c mice treated with AMP + NEO had decreased permeability compared to BALB/c controls (permutation *t* test: *p* < 0.05). When comparing FITC recovery between strains within treatment groups, BALB/c mice tended to have increased permeability compared to C57BL/6 in both the control group (permutation *t* test: *p* < 0.1) and after ERY (permutation *t* test: *p* < 0.1).

### Taxa correlations with small intestine permeability

AMP + NEO treatment affected BALB/c and C57BL/6 microbiota compositions in a similar manner, consistently eliminating many of the same taxa, with only a few taxa significantly responding to treatment in a mouse strain-dependent manner. To consider how specific taxa may influence permeability, we explored the differential responding taxa in relation to barrier permeability and revealed a significant positive correlation with FITC recovery in BALB/c mice with unclassified *Peptococcaceae* (Spearman’s *ρ* = 0.73, FDR < 5%). Other significant positive correlations (*p* < 0.05) were found with *Lachnospiraceae* (Spearman’s *ρ* = 0.59, FDR < 15%) *Clostridium*, unidentified Lachnospiraceae (Spearman’s *ρ* = 0.53, FDR < 15%), unclassified Ruminococcaceae (Spearman’s *ρ* = 0.51, FDR < 15%) and unclassified Bacteroidales (Spearman’s *ρ* = 0.51, FDR < 15%), along with a negative correlation with Bacteroidaceae *Bacteroides* (Spearman’s *ρ* = − 0.57, FDR < 15%), though none of these effects remained significant after correcting for multiple comparisons (Suppl. Fig. 2). No significant correlations were found in C57BL/6 mice. Interestingly, *Parabacteroides* and unclassified *Alcaligenaceae* both increased in response to AMP + NEO in C57BL/6 mice only and may account for differential shifts in FITC recovery observed between these two mouse strains in response to AMP + NEO. Positive correlations with FITC *z* score values were found following AMP + NEO treatment with *Parabacteroides* (Spearman’s *ρ* = 0.90, FDR < 1%), an *Alcaligenaceae* OTU, (Spearman’s *ρ* = 0.80, FDR < 5%), *Catabacteriaceae* (Spearman’s *ρ* = 0.78, FDR < 5%), and an unclassified Bacteroidales OTU (Spearman’s *ρ* = 0.77, FDR < 5%). A negative correlation was also found between FITC and Bacteroidaceae *Bacteroides* (Spearman’s *ρ* = − 0.75, FDR < 5%). No significant correlations were found in ERY-treated mice (Fig. 4d).

### Antibiotics treatment AMP + NEO differentially effected tight junction expression

**Fig. 5:**
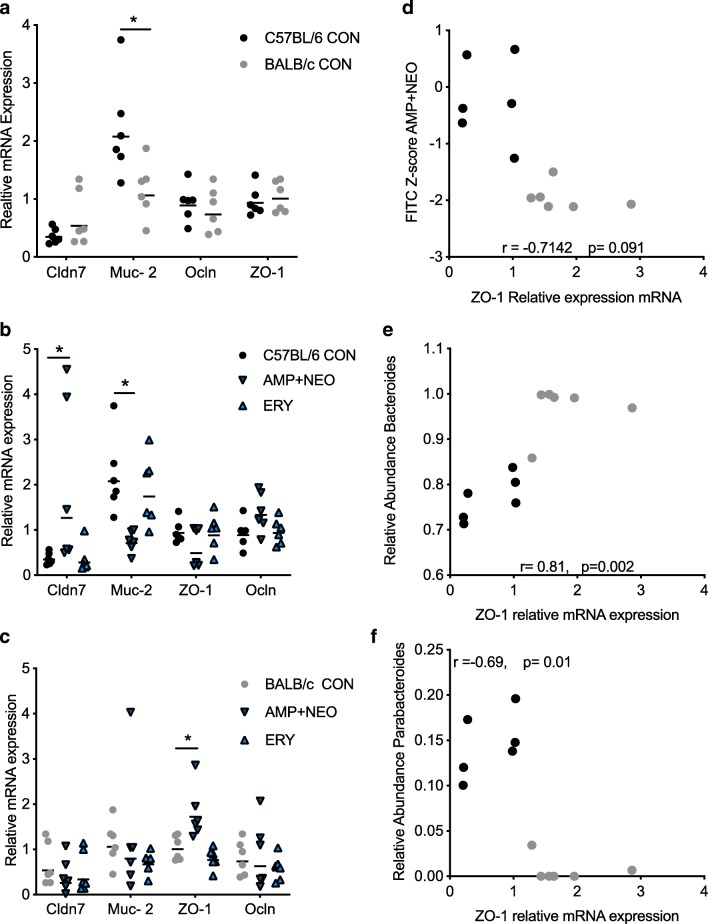
mRNA expression of intestinal barrier regulating genes. **a** Small intestine claudin 7 (Cldn7), mucin 2 (Muc-2), occludin (OCLN), and zonuline-1 (ZO-1) mRNA expression normalized to GAPDH and β-actin gene relative expression in C57BL/6 and BALB/c control groups (muc-2: *p* < 0.05). **b** C57BL/6 mRNA expression post treatment AMP + NEO and ERY (Cldn7: *p* < 0.01, Muc-2: *p* < 0.01). **c** BALB/c mRNA expression post AMP + NEO and ERY (ZO-1: *p* < 0.05). **d** Correlation analysis of small intestinal barrier permeability post AMP + NEO treatment with expression of ZO-1 mRNA (*r* = − 0.71). **e**, **f** Spearman’s correlation between relative abundance of the genera *Bacteroides* (*r* = 0.75) and *Parabacteroides* (*r* = − 0.65) and the relative expression of ZO-1, post treatment AMP + NEO in both BALB/c and C57BL/6 mice

To determine if alterations in small intestine permeability were associated with changes in expression of tight junctions and mucin, RT-qPCR on RNA isolated from sections of ileum tissue collected post-FITC gavage was performed (Fig. 5a–c). Within the control groups, a significant difference in the expression of mucin-producing gene Muc-2 mRNA was observed with C57BL/6 mice exhibiting an increase in relative expression compared to BALB/c mice (Fig. 4a). Significant increases in relative expression of in the tight junction protein Claudin 7 mRNA (Cldn7) were observed in C57BL/6 mice but not in BALB/c mice after AMP + NEO compared to control group, as well as a decrease in Muc-2 mRNA expression (Fig. 5b). Despite significant alterations to Cldn7 mRNA and Muc-2 mRNA expression, there was no significant correlation to FITC recovery in C57BL/6 AMP + NEO-treated mice and gene expression, indicating these genes may have additional roles outside of barrier tightening. A significant increase in tight junction scaffolding protein ZO-1 was found in response to AMP + NEO treatment only in BALB/c mice. A negative correlation (Spearman’s *ρ* = − 0.71, *p* > 0.05) was found with the expression of ZO-1 mRNA and FITC *z* score following AMP + NEO treatment (Fig. 5d), indicating that increased expression of ZO-1 mRNA correlated with a reduction of small intestine permeability. An analysis on the taxa that previously were shown to correlate with FITC *z* scores confirms the relationship between the taxa and barrier function with *Parabacteroides* and *Alcaligenaceae* relative abundance’s negatively correlating with ZO-1 mRNA expression in AMP + NEO-treated BALB/c and C57BL/6 mice (Spearman’s *ρ* = − 0.9, *p* < 0.01, *ρ* = 0.8 *p* < 0.01 respectively). A positive correlation with relative abundances of Bacteroidaceae *Bacteroides* and ZO-1 mRNA expression was found in response to AMP + NEO treatment (Spearman’s *ρ* = 0.75, *p* < 0.01 (Fig. 4f). No significant differences in tight junction expression or Muc-2 mRNAs were found for ERY treatment in either mouse strains.

## Discussion

Exploring the gut microbiota composition of BALB/c and C57BL/6 mice revealed distinct differences in composition. Since experimental mice were obtained from two different sources and housed in pairs of the same strain, the observed differences can be attributed in part to host mouse strain and in part to environmental factors such as source and co-caging conditions (Benson et al. 2010; Brinkman et al. 2013; Campbell et al. 2012; Choo et al. 2017; Deloris et al. 2006; Ericsson et al. 2015; Hoy et al. 2015; Kovacs et al. 2011; O’Connor et al. 2014). The new findings here show that specific taxa influence gut barrier integrity and permeability. Microbe-host interactions at the gut barrier influence inflammation and are an important component of the microbiota-immune-brain axis. At the genus level, group differences were observed in the relative abundance of genera *Prevotella*, *Alistipes*, and *Akkermansia*, with a significantly higher relative abundance of *Alistipes* found in BALB/c and while higher relative abundance of *Prevotella* and *Akkermansia* were found in C57BL/6 mice. These results are in accordance with previous studies focused on host genetic influence on murine gut microbial compositions (Hildebrand et al. 2013; Krych et al. 2013; Xiao et al. 2015). The differences in relative abundance of *Prevotella* are of interest as *Prevotella* is one of the defining taxa associated with human enterotypes (Arumugam et al. 2011; de Moraes et al. 2017; Wu et al. 2011). Previous work has also identified the relative abundance of *Alistipes* to be under the control of a quantitative trait loci (QTL) in mice, suggesting a role of host genetics in its relative abundance (Leamy et al. 2014). Interestingly, the genus *Akkermansia* has been shown to be found in mice of C57BL/6 background (Fransen et al. 2015) and responsive to a variety of other factors including diet. As our mice had a common diet, this is unlikely to have contributed to the differences observed, more likely it is a combination of host genetics and housing, as differences in abundance levels from different suppliers has been shown as well as changes in *Akkermansia* levels over time in animals from the same facility (Choo et al. 2017; Ericsson et al. 2015; Hoy et al. 2015). The higher abundances of *Akkermanisa* in C57Bl/6 may be due to increased availability of niche energy source Muc-2 in C567Bl/6, as a previous study indicated that *Akkermansia muciniphila* has the ability to degrade Muc-2 O-glycans in vitro (Png et al. 2010). The increased expression of Muc-2 mRNA in C57BL/6 mice observed here may provide a niche environment for these bacteria to thrive.

Our results indicate that perturbation of bacterial composition with broad-spectrum antibiotics altered barrier integrity and function in a taxa-dependent and taxa-independent manner. It is important to link specific taxa to host physiology and to identify which taxa are responsive to perturbation. Previous studies have evaluated differences between BALB/c and C57BL/6 mice at the gastrointestinal barrier (Volynets et al. 2016a), specifically finding differences in IgA production (Fransen et al. 2015), and the expression of antimicrobial peptides (Volynets et al. 2016b). Previous work in rats has shown that changes in gastrointestinal permeability may be linked to different antibiotic classes, which may account for the lack of significant change with erythromycin treatment observed here (Tulstrup et al. 2015). Our analysis of alterations of microbial composition at the genus level in response to antibiotic treatment revealed significant treatment by strain interactions for *Parabacteroides* and a *Peptococcaceae* OTU. *Parabacteroides distasonis* has been shown to carry ampicillin resistance genes (Nakano et al. 2011), and while our analysis was not resolved to the species level, it is possible that the presence of ampicillin resistance genes may account for overgrowth of the *Parabacteroides* genus in C57BL/6 mice. A significant association of barrier permeability and the relative abundance of *Parabacteroides* was found, with an increase in *Parabacteroides* correlating to increased barrier permeability. The increase in *Parabacteroides* in C57BL/6 group in response to antibiotics may be counteracting antibiotic-mediated tightening of the gastrointestinal barrier. A role for *Parabacteroides*-host signaling influencing intestinal barrier integrity was further supported by a significant negative correlation between *Parabacteroides* and expression of barrier regulating ZO-1 mRNA. BALB/C mice exhibited a host-specific change in expression of ZO-1 mRNA, a universally expressed tight junction adaptor protein (Volynets et al. 2016a). These results confirm previous work demonstrating ZO-1 expression correlates to intestinal permeability, with the expression of ZO-1 in both C57BL/6 and BALB/c mice post treatment significantly correlating to changes in small intestinal permeability (Volynets et al. 2016a). In addition to *Parabacteroides*, an association between the relative abundance of genus *Bacteroides* and barrier permeability was observed, with *Bacteroides* abundance exhibiting a positive impact on barrier function. The results here support previous reports of *Bacteroides* sp. increasing barrier function (Hooper et al. 2001; Hsiao et al. 2013). Notably, Hsiao and colleagues demonstrated the clinical relevance of the probiotic *Bacteroides fragilis*, which was found to restore alterations in barrier function, microbiota composition, and reduced behavioral defects in a mouse model of autism spectrum disorder. Importantly, these key taxa identified here that are linked to antibiotic-related alterations to the gut microbiota composition may be important mediators of intestinal permeability.

Differential responses to antibiotic treatment were found in the mRNA expression of barrier regulating tight junction protein Cldn7 in response to AMP + NEO only in C57BL/6 mice. These subtle changes in gene expression are not surprising as the exposure period in this study was 2 weeks. It is likely that longer exposure to antibiotics would lead to greater changes in host gene expression and barrier function. The increased expression of Cldn7 mRNA in C57BL/6 mice is of interest since claudins are known to play critical structural and functional roles in tight junction complexes that moderate epithelial permeability (Van Itallie and Anderson 2006). In the current work, Cldn7 mRNA increased independent of changes in intestinal permeability and was not associated with specific bacterial taxa. Recent evidence has shown that Cldn7 has additional roles maintaining intestinal homeostasis (Ding et al. 2012). Cldn7 is expressed on a basolateral membrane and interacts critically with extracellular matrix (ECM) components, with loss of function resulting in inflammation with IBS like morphology changes and uncontrolled remodeling of ECM (Ding et al. 2012). The increase in Cldn7 mRNA expression may be indicative of alterations in ECM composition or immune signaling cascades as well part of the host-specific response to maintaining intestinal homeostasis under altered microbial conditions.

A bidirectional relationship between host miRNA expression and gut microbiota composition has been reported (Dalmasso et al. 2011; Liu et al. 2016; Singh et al. 2012), demonstrating a role for microbiota in regulating host miRNA expression in intestinal epithelial cells (IEC) and on bacterial diversity (Liu et al. 2016). Exploring the functional eukaryotic gene targets of the differentially expressed fecal miRNA revealed targets genes localized to the mucin O-type biosynthesis pathway, the end products of which make up the mucus layer of the gastrointestinal tract. Differences in mucus layer organization and composition have been previously shown to impact gastrointestinal microbiota, as the glycosylated mucins provide a unique energy source to mucolytic bacteria (Staubach et al. 2012). Differences in mucus layer thickness have been shown to affect secretory immunoglobulin (sIgA) distribution (Johansson et al. 2009), a factor known to play a critical role in maintaining intestinal homeostasis (Corthesy 2013). The functional pathway analysis of the top expressed fecal miRNA yielded similar significant pathways involving complex glycoprotein synthesis, with targets in the extracellular matrix, mucus O-glycan biosynthesis, and proteoglycans pathways. Interestingly, many of the eukaryotic glycan processing genes targeted by the fecal miRNA have prokaryotic homologs (El Kaoutari et al. 2013).

Evaluating the potential for eukaryotic host miRNA to target bacterial genes is a recent consideration and the published work provided evidence of potential regulation of bacterial composition by miRNA (Liu et al. 2016; Moloney et al. 2018; Teng et al. 2018). Previous work looking at bacterial sRNA gene regulation have found multiple possible binding sites for bacterial sRNA to bacterial genes (De Lay et al. 2013) and understanding the role of miRNA in host-microbe interactions is needed. Our current results are in parallel with that of others that demonstrated the presence of host-derived miRNAs in murine (Liu et al. 2016; Moloney et al. 2018) and human feces (Liu et al. 2016). Reduction of host fecal miRNAs has been shown to alter gut bacteria composition and influence bacterial growth (Liu et al. 2016; Moloney et al. 2018). The potential for miRNAs to enter bacterial cells was shown by Liu et al. (2016) and more recently, evidence demonstrated that plant-derived exosomal miRNAs are taken up by gut microbiota and target bacterial genes to influence cross-talk between gut microbiota and the host immune system (Teng et al. 2018). Interestingly, specific targeting of food-derived exosomal miRNAs to mucosal-associated bacteria, *Lactobacillus*, was highlighted (Teng et al. 2018). Of those taxa at genus and family level that were conserved within our dataset, 14 significant correlations between fecal miRNA counts and the relative abundance of the predicted taxa were found. Most notably we found a strong negative correlation with let-7b-5p and the relative abundance of *Parabacteroides*. let-7 family miRNA expression has previously been shown to be involved in the host anti-bacterial defense through modulation of immune response (Schulte et al. 2011). Intriguingly, *Parabacteroides* has also been associated with inflammatory markers (Conley et al. 2016) and here has been found to respond to antibiotic treatment. We also found a positive association between mir-21a-5p and the relative abundance of predicted genus *Akkermansia*. *A. muciniphila* has been previously shown to play a role in energy metabolism, showing a role in regulating adiposity, by regulating mRNA expression of markers of adipocyte differentiation and lipid oxidation (Everard et al. 2013). Mir-21a-5p has also been shown to play a critical role in regulating the proliferation of human adipose tissue (Kim et al. 2012); thus, the correlation observed here maybe reflective a mechanistic link between *Akkermansia* abundance and miR-21 expression in energy metabolism of the host. *Prevotella* was found to be one of the most divergent taxa between groups, interesting miR-2134 which predicted to regulate *Prevotella* classified gene based on BLASTn analysis negative correlated with *Prevotella* abundance. As previously mentioned, *Prevotella* abundance contributes to known human enterotypes, while much work has focused on the diet aspect of enterotypes (Wu et al. 2011), results here now suggest that host-microbe communication may also contribute. Several negative correlations were found with miRNA predicted to affect Clostridiacea *Clostridium*. The need for the host to regulate clostridium may relate to the numerous *Clostridium* spp. that can be pathogenic, or due to *Clostridium*’s role in modulating host physiology and homeostasis by controlling regulatory T cell development and producing barrier modifying SCFA (Smith et al. 2013). It is likely that the targeting of these bacterial genes could affect bacterial growth; this suggestion is supported by the current analysis as well as the work of Liu et al. (2016), who found increase in bacterial growth, after miRNA targeting. In addition to the four positive correlations we found between fecal miRNA and predicted bacterial targets relative abundance, we also observed negative correlations between miRNA expression and relative abundance of these taxa, which indicate a potential regulator effect ton bacterial growth; however, these relationships require further in vivo investigation.

## Conclusions

The results here provide useful insight into the mechanisms involved in host-microbe interactions. The collective work on understanding the roles of intestinal based miRNAs has revealed their potential to act as a regulator of gut homeostasis. Understanding the complex relationship between the host and its gut microbiota and the mechanisms that mediate its homeostasis is of critical importance to this rapidly developing field. Here, we show differences in two inbred strains of mice; however, the vendor sources were different and therefore both gene and environmental factors are contributing to our results. Importantly, we show additional evidence about how the host influences gut microbiota composition and how manipulating microbiota composition impacts barrier integrity and function, as well as provide preliminary evidence of a potential mechanism of host miRNA-microbe interactions.

## Electronic supplementary material

ESM 1 (PDF 500 kb)

ESM 2 (XLSX 27 kb)

ESM 3 (XLSX 130 kb)
