# Supplementary material for: Microbe and host interaction in gastrointestinal homeostasis
Source: Psychopharmacology (Berl). 2019 Mar 21;236(5):1623–40. doi: 10.1007/s00213-019-05218-y (PMC6599184; doi:10.1007/s00213-019-05218-y)
Supplement: Supplementary file 1 — (PDF 500 kb) [file 213_2019_5218_MOESM1_ESM.pdf]

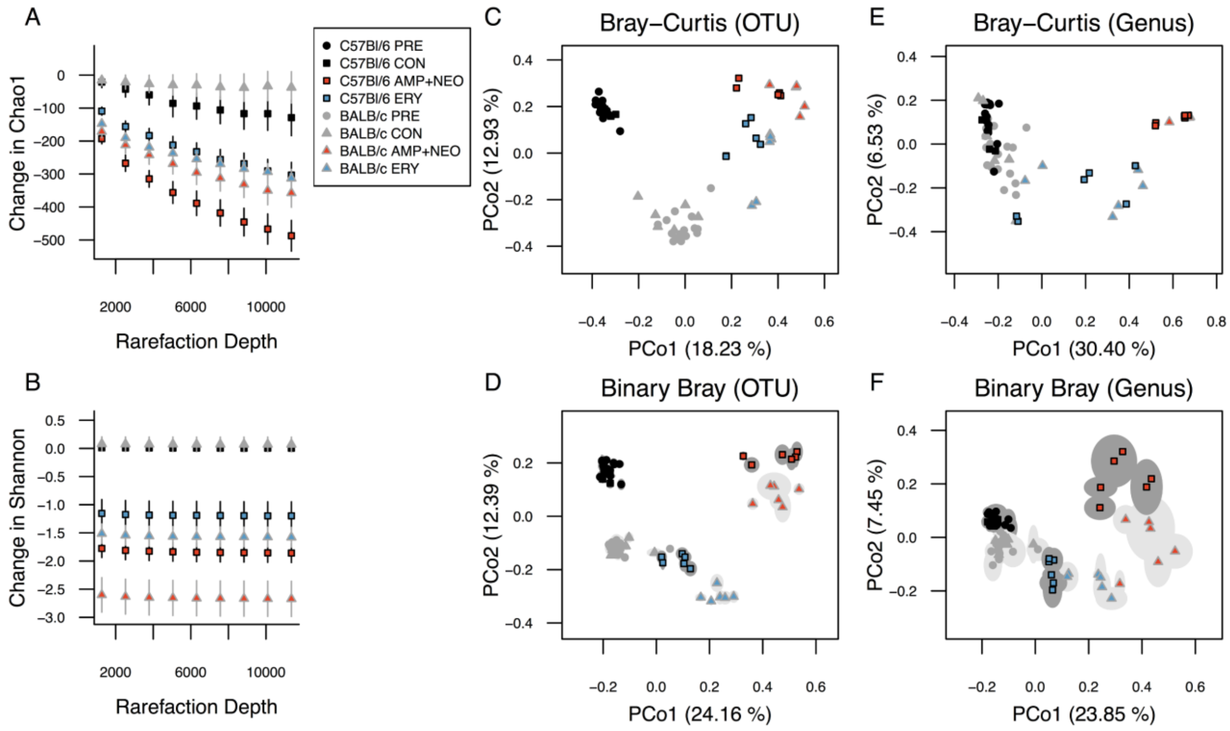

Supplemental Figure 1. Effect of antibiotic treatment on Alpha and Beta Diversity. (A) Change in alpha diversity post treatment with AMP+NEO and ERY in BALB/c and C57Bl/6 as measure by Chao1 index, and (B) Shannon index. (C-E) Effect of antibiotic treatment on beta diversity, as measured by Bray-Curtis for both OTU and relative abundance genus level. (D-F) Effect of antibiotic treatment on Beta Diversity as measured by the binary Bray-Curtis at the OTU and genus level.

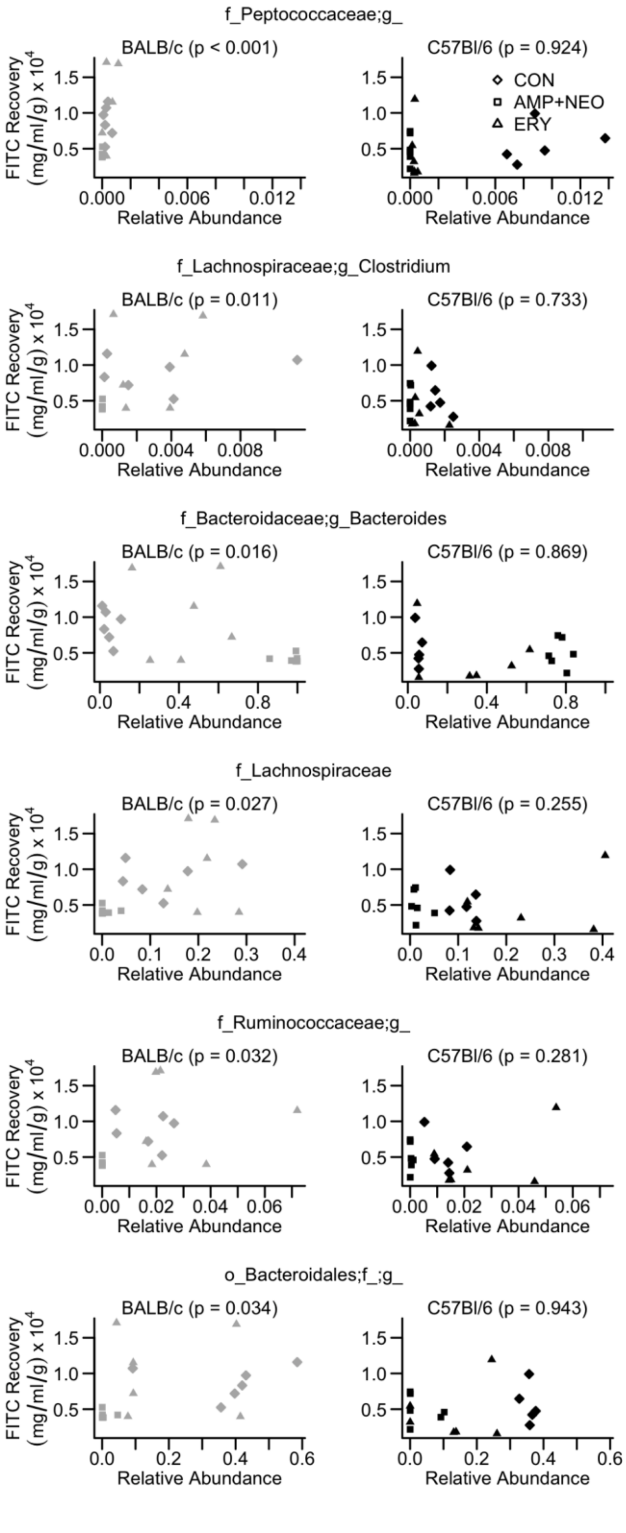

Supplemental Figure 2. Spearmen's rank correlation analysis between differentially antibiotic responding taxa as identified in Figure 4C and small intestinal barrier permeability as measured by FITC recovery.
